# Editorial: The new DSM is coming – it needs tough love …

**DOI:** 10.1111/jcpp.12078

**Published:** 2013-04-12

In soulless conference rooms of East Coast hotels and long-winded telephone meetings, the new DSM was conceived. It is not very different to the DSM-IV we all know, more its tweaked clone (spot the Arabic numeral that has replaced the Latin digits). Yet, its birth is anything but a dull event: the DSM-5 is suffering perinatal stress from the criticisms of scientists, clinicians and the public.

The very scale of the DSM project (http://www.dsm5.org) may explain some of the highly critical reception. The DSM-5 is meant to be useful to clinicians, avoid public health disasters, satisfy health care providers, have an international appeal, and at the same time, do justice to science. Inevitably, such a project will contain contradictions, imperfections and go against some preference or interest of those it is meant to serve.

For example, it is no surprise that scientists are sceptical of the DSM-5. Many of the categories psychiatrists have been using for decades are problematic: we have difficulties separating diagnoses from each other (evidenced by high rates of overlap between them), we know their aetiology only in gross terms (e.g. we know that bipolar disorder is highly heritable, but do not know enough about the genes that cause it), and we are just beginning to understand their pathophysiological processes (e.g., that the cortex may mature more slowly in ADHD) (Shaw et al., 2012). Moreover, some of the classifications seem arbitrary, such as the separation between personality disorders from the rest of psychiatry^2^. The new DSM (American Psychiatric Association, 2013) is highly unlikely to solve these problems and it is precisely the job of scientists to criticize these shortcomings. It is also right for scientists to want to be radical and prefer research innovation over some of the stale consensus upon which nosology is built. Yet, as can be true of radicals, scientists often have little more than a futuristic vision to offer as an alternative. The great hope that either genes or neuroimaging would become clinically useful biomarkers has not yet been fulfilled. Moreover, the vehement calls to “move beyond” descriptive phenomenology (the basis of current nosology) ignores that even in those medical disciplines where biomarkers are a big success (e.g. oncology), clinical description is still indispensable.

The DSM also has to consider public health, alongside clinical and research goals. The introduction of the category of disruptive mood dysregulation disorder (DMDD) is a good example of the impossible position nosologists find themselves in. A few years ago, psychiatrists, paediatricians and the US public discovered that bipolar disorder was being diagnosed at dramatically higher rates (up to 500% more) than it used to be. The public health implications were vast (a similarly sharp rise in antipsychotic prescription rates was documented for the same time period). Research findings suggested that the staggering increase might be because children with severe irritability were being mislabelled as suffering from bipolar (Leibenluft, 2011). While severely irritable children were not necessarily bipolar, they also did not fit neatly into any other psychiatric diagnosis and were aptly described as diagnostic orphans. The DSM-5 deals with this by proposing the new DMDD category precisely to accommodate such children and to avoid labelling and treating them as if they were manic. The APA acted out of a good motivation and, had they done nothing, they might have been accused of complacency. Having now introduced DMDD, the DSM has attracted some hefty comments. Some of the criticism is fairly general and applies to DMDD as much as to any other psychiatric category: irritability could be thought more of as a dimension than a category, more research is needed into the validity of DMDD, and its boundaries with other disorders should be clarified (Krebs et al., 2012). Only time will tell whether the additional criticism that DMDD will pathologize normal children's tantrums is credible – results so far (Copeland, Angold, Costello & Egger, 2013) suggest that DMDD is not that common. The example illustrates that to many eyes, the DSM may be damned if it does and damned if it does not.

What are the alternatives to the current DSM-5?

One possibility could be for the DSM only to classify disorders of well-known aetiology. This would mean focusing on the relatively rare monogenetic disorders and some of the dementias, and leave out the vast majority of mental illness. This might satisfy purists, but would do a disservice to patients and probably stifle progress in psychiatry. It would be more pragmatic to use such well-defined disorders as prototypes. The DSM could cut its teeth with them in preparation of more clinically useful biomarkers for other disorders in future. For instance, it could try to figure out how to deal with heterogeneity in outcome among people with the same genetic disorder.

Another possibility is for the DSM to take a more haughty approach and not get embroiled in public health debates (such as the bipolar controversy discussed above). This might look convenient, but it would strongly diminish nosology's relevance for the real world. Instead of fleeing reality, the APA should do more to confront it and invest in large, well-designed field trials.

Yet another option is for the DSM to become smaller. Instead of trying to balance science, public health and clinical needs, it should limit itself to being something of a brochure for clinicians. Morphological classifications of tumours or the grouping of peripheral nerve lesions are some examples. This approach would be dull and would also undo years of progress in mental illness. Instead, the DSM could consider allowing several levels of classification, each with a more circumscribed aim. One level could be useful for first-line clinicians and include symptomatology and clinical predictors of treatment response; another level would be of relevance to diagnosticians at tertiary centres and be based on, say, neural activity in the subgenual anterior cingulate cortex. Working out the overlap between such levels of description could lead to refreshingly new research. The most obvious first step to such an approach (and one long overdue) is to integrate dimensions into the diagnostic system. We all know that many psychiatric disorders (ADHD being a leading example) are best thought of as dimensions and that imposing an arbitrary cut-off to generate diagnoses is problematic. Instead, thresholds can be empirically determined according to purpose: clinical cut-offs will often differ from those useful for aetiological research or preventive interventions.

Whatever we do and no matter how good the new DSM will prove to be, there is clearly a lot of work ahead. This Journal has a long tradition of publishing the research required to build a new nosology (Rutter, 2011). Take this issue's three articles about ADHD as an example that covers the ground from pathophysiology right through to treatment. *Karalunas* et al.^1^ challenge the long-held notion that intra-individual variability in ADHD is mainly present in low frequencies and contributes to our understanding of disease mechanisms. *Chen* et al. use epidemiological data to report that the overlap of ADHD with tics is associated with a higher risk of allergy than either ADHD or tics alone, a clear demonstration of how embedded psychiatry is within the rest of medicine. *Simonoff* et al. venture into territory that others have long feared to tread – they present data from the first longer term (16 weeks) randomized, double-blind, placebo-controlled trial of methylphenidate in young people with ADHD and intellectual disability. The results have key implications for this traditionally under-served population of children and, in addition, the results raise important questions for nosologists. We may be too rigid about IQ-based distinctions and too slow to recognize that dimensions (such as treatment responsiveness) may cut across the boundaries of intellectual disability.

So, what can we do to nurture the newly born nosology? A few things: criticize it; suggest changes; do more research (after all, classification is on the uphill path from bench to bedside); and more importantly, show the DSM the love – the tough love – it deserves.

## Notes

1 Articles in this issue (with italicized names on first mention) can be found at: http://onlinelibrary.wiley.com/doi/10.1.1.1.1/jccp.2013.54.issue-5/issuetoc.

2 See also the editorial and articles published in the 2012 JCPP Annual Research Review: Nosology in developmental psychopathology: DSM-5, ICD-11 and beyond, available at: http://onlinelibrary.wiley.com/doi/10.1111/jcpp.2012.53.issue-5/issuetoc
